# Effects of Water Addition on Reproductive Allocation of Dominant Plant Species in Inner Mongolia Steppe

**DOI:** 10.3389/fpls.2020.555743

**Published:** 2020-11-16

**Authors:** Yongjie Liu, Zhenqing Li

**Affiliations:** ^1^State Key Laboratory of Grassland Agro-ecosystems, Key Laboratory of Grassland Livestock Industry Innovation, Ministry of Agriculture and Rural Affairs, College of Pastoral Agriculture Science and Technology, Lanzhou University, Lanzhou, China; ^2^State Key Laboratory of Vegetation and Environmental Change, Institute of Botany, Chinese Academy of Sciences, Beijing, China; ^3^University of Chinese Academy of Sciences, Beijing, China

**Keywords:** biomass, climate change, reproductive allocation, vegetative biomass, water availability

## Abstract

Extreme events such as extreme drought and precipitation are expected to increase in intensity and/or duration in the face of climate change. Such changes significantly affect plant productivity and the biomass allocation between reproductive and vegetation organs (i.e., reproductive allocation). Our aims are to test the effects of water addition on the trade-offs in allocation of plant biomass and whether such effects are modified by species. A manipulative experiment was conducted from May 2000 to October 2001, where four dominant plant species (i.e., *Leymus chinensis*, *Stipa grandis*, *Artemisia frigida*, and *Potentilla acaulis*) in the Inner Mongolia steppe in China were treated with 8 levels of water addition. Results demonstrated that water addition significantly affected the reproductive allocation of plants, and such effects were modified by species. Specifically, with increasing water availability, *L. chinensis* was not impacted, while *A. frigida* allocated more biomass to reproductive organs than to vegetative organs, while such allocation in *S. grandis* and *P. acaulis* first decreased, and then increased after reaching a peak. Our results indicated that plant species can adjust their reproductive allocation patterns to deal with water availability gradients. Climatic factors such as rainfall and temperature usually co-appearing, thus future research should explore the joint effects of several climate change factors on grasslands in order to maintain the health and sustainability of grasslands.

## Introduction

Climate change crucially impacts the dynamics of plant individuals, populations and ecosystems (Jentsch et al., 2007; Hoover et al., 2014; Estiarte et al., 2016; Ryalls et al., 2016; Collins et al., 2017). Rainfall, as one of the main climatic factors, considerably impacts plant growth (Thomey et al., 2011; Gao et al., 2015). Extreme drought and rainfall events are predicted to vary spatially and temporally under climate change (IPCC, 2007; Benestad et al., 2012). The range of rainfall amount impacts terrestrial ecosystems as water limits plant growth, reproduction and productivity (Koerner et al., 2014; Estiarte et al., 2016; Felton et al., 2019). Biomass is an important variable that can be applied to explore the response of plants to the changes of rainfall. Studies found that plants could allocate more biomass to the organs that need to acquire more resources according to the optimal allocation theory (Bloom et al., 1985; Gedroc et al., 1996). Thus, biomass allocation is a key strategy for plants to deal with climate change (Harper and Ogden, 1970; Mokany et al., 2006). Reproductive allocation refers to biomass allocation to reproductive organs of plants relative to total biomass (Harper and Ogden, 1970; Weiner, 2004). Many studies have explored the effects of climate change on biomass allocation of plants (Wilson and Thompson, 1989; Fay et al., 2000; Simon et al., 2007; Brenes-Arguedas et al., 2013). However, most of these studies only focus on aboveground biomass (Achten et al., 2010; Liu et al., 2015), and fewer have considered the above/belowground allocations, and even fewer considered the biomass allocations between reproductive and non-reproductive organs. Thus, a gap in knowledge exists on the reproductive allocation between these organs of plants (Kreyling et al., 2014).

Abiotic factors such as temperature, precipitation and nutrients affect the reproductive allocation of plants (Guo et al., 2010; Cheplick, 2020). Wilson and Thompson (1989) found that the growth form of plant species affected their reproductive allocation, where species with a rhizomatous or stoloniferous had low values of reproductive allocation. However, some studies found that resources (Guo et al., 2010) not growth form (Schat et al., 1989) determined the reproductive allocation, where increasing nitrogen and potassium addition improved the reproductive allocation of *Leymus chinensis*, while increasing phosphorus addition had no influence on the reproductive allocation of this species (Guo et al., 2010). Niu et al. (2006) found that fertilizer addition can increase the reproductive allocation of herbs, but not non-herb species. However, the underlying mechanisms in these patterns are still far from clear. Therefore, the reproductive allocation of plants merits further research.

As one of the main terrestrial ecosystems, grasslands occupy more than 30% of the terrestrial area (Parton et al., 2012), and they are important for biodiversity, economics, biogeochemical cycles and energy transformation (Huang et al., 2010; Bai et al., 2012). Grasslands are sensitive to climate change compared with the forests (IPCC, 2003; Gherardi and Sala, 2015; Eziz et al., 2017; Maurer et al., 2020). Although there is no consistent conclusion on the climate change rate, range and area, the general pattern is that it will be moister in the south part and drier in the north part of China (Zhao et al., 2013). Some studies explored the effects of rainfall changes on plant dynamics (Simon et al., 2007; Yang et al., 2008; Brenes-Arguedas et al., 2013; Wilcox et al., 2017; Gao et al., 2019; Zhou et al., 2020). However, these studies found contrasting results in plants’ morphological traits, resource allocation and distribution (Heisler-White et al., 2009; Wilcox et al., 2015), and the impacts of the rainfall amount on reproductive allocation of plants are still unclear.

To explore the effects of water availability on reproductive allocation of plants under climate change, an experiment was conducted by applying eight levels of water addition to four dominant plant species [i.e., *Leymus chinensis* (Trin.) Tzvel., *Stipa grandis* P.A. Smirn., *Artemisia frigida* Willd., and *Potentilla acaulis* L.] in the Inner Mongolia steppe in China, where *L. chinensis* is a perennial forage grass with long strong rhizomes, and *S. grandis* is a perennial tussock grass with closely clumped shoots, while *A. frigida* and *P. acaulis* are perennial herbs with stolons and developed adventitious roots (Li et al., 2005; Liu et al., 2006).

Species with rhizomes and stolons tend to have low reproductive values (Wilson and Thompson, 1989), so our first hypothesis was that *L. chinensis, A. frigida* and *P. acaulis* have smaller reproductive values than *S. grandis*. Moreover, Niu et al. (2006) revealed that the reproductive allocation could be modified by the resource amount. Therefore, our second hypothesis was that the reproductive allocation value of *L. chinensis, A. frigida*, and *P. acaulis* may increase with water availability, while a different pattern may occur in *S. grandis.* This study will shed light on improving our understanding of the reproductive allocation of plants in response to a gradient of water availability. Exploring the responses of dominant species to water availability is valuable for grassland persistence and rangeland sustainability.

## Materials and Methods

### Study Field

This experiment was conducted on the Inner Mongolia steppe (43°33′N, 116°40′E), with elevation ranging from 1,200 to 1,250 m (Bai et al., 2004, 2012). This site is characterized by a humid summer and dry winter. The mean annual temperature is around −1.1°C, where the coldest month is about −21.4°C in January, while the warmest month is 18.5°C in July. The frost-free period is around 100 days in a year. The mean annual precipitation between 1980 and 2000 is 350 mm, where the rainfall mainly falls between June and August, and the amount of rainfall during this period presents around 80% of the annual rainfall (Jia et al., 2005; Bai et al., 2007).

### Experimental Design

**FIGURE 1:**
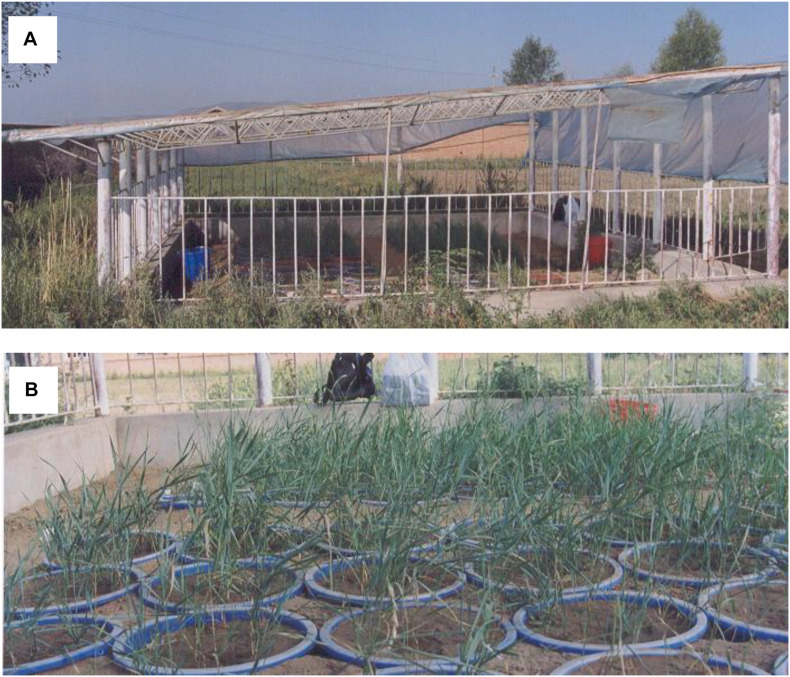
The experimental setup **(A)** and inside of the experiment **(B)**.

To explore the responses of plants to a gradient in water availability, a manipulative experiment was conducted from May 2000 to October 2001, where four dominant grasses species in the Inner Mongolia steppe (*L. chinensis*, *S. grandis*, *A. frigida*, and *P. acaulis*) were treated with eight levels of water addition, which were selected based on the natural variation around the mean annual precipitation in the study field (i.e., 350 mm during 1980–2000, Supplementary Figure S1). Thus, the eight levels of water addition were 170, 250, 300, 350 mm (i.e., the basic level of total water amount), 525, 595, 665, and 700 mm (Table 1). Such a large range of water availability gradient, especially the extreme drought and extreme wet conditions, was set to investigate the responses of the grasses species to the change of water availability facing climate change. To remove effects of natural rainfall, this experiment was conducted in a plot with a temporary rainout shelters (Power et al., 2016). The shelter was covered upward from 2 m above the ground with highly transparent plastic foil to allow wind circulation and prevent warming (Figure 1).

**TABLE 1:** Eight levels of water addition in this experiment.

Treatments	T1	T2	T3	T4	T5	T6	T7	T8
Water addition (%)	−50%	−30%	−15%	0	+ 50%	+70%	+ 90%	+100%
Water amount (mm)	170	250	300	350	525	595	665	700

Plants were grown in individual pots (30 cm in diameter, 50 cm in height, Poorter et al., 2012) with soil collected from the nearby grasslands, where only the topsoil to 50 cm depth was collected and well mixed, and litters and roots were carefully removed. Note that the collected soil is mainly dark chestnut soil, and the humus layer is thin. All pots were dug into the ground, and surface of the pots were kept at the same level with the nearby soil surface, where four holes with 10 mm-diameter in the bottom of each pot ensured drainage of water. For *L. chinensis* and *S. grandis*, seeds were sown in three pots for each species in early May 2000, and four individuals with the visually similar size were kept in each pot after germination, and the remaining seedlings were removed manually. However, rate of *S. grandis* was too low, so four ramets with visually similar size from the nearby field were transplanted into each pot in later May 2000. For *A. frigida* and *P. acaulis*, plants were first dug out from the nearby field, and then four ramets with visually similar size were transplanted into each pot in early May 2001. All plants grew in an open air area without rainout shelter before applying water addition, which happened from 10 June 2001 to 10 September 2001. During the experiment, water was simply added daily instead of following the local rainfall events since the original aim was to explore the general response trend of plants to a gradient of water availability. For the same reason, soil water contents in these pots were not measured. The amount of water applied in each treatment was calculated by dividing the total amount of water by the experimental period. Water was evenly added by hand using a hose with a shower in order to not make water runoff at the soil surface.

By the end of the experiment, almost all plants set seeds. At the end of the experiment (in the middle of September 2001), all plants in each pot were washed out from their growing soils, and biomass was separated into two groups, i.e., reproductive organs and vegetative organs, where the vegetative organs include leaves, stem and roots, while the reproductive organs include flowers, rhizomes and stolons. Thus, the vegetative organs and reproductive organs of the four species are: *L. chinensis* (rhizome; leaves + stem + roots), *S. grandis* (seeds; roots + stem), *A. frigida* (flowers; leaves + stems + roots), and *P. acaulis* (stolon; leaves + roots). They were oven-dried at 65°C to constant weight and weighted.

### Statistical Analysis

Total biomass of each species was calculated by summing up reproductive biomass and vegetative biomass in each pot, and then converted to g m^–2^ through dividing by the pot surface area; and ratio of reproductive biomass to total biomass (R: T) of each species was calculated by dividing the reproductive biomass by total biomass.

Two-way analysis of variance (ANOVA) was conducted to investigate the effects of species, water addition and their interaction on the total biomass, reproductive biomass, vegetative biomass, and R: T ratio. *Post hoc* analysis (pairwise comparisons with Bonferroni corrections) was used to test for differences between the grasses species.

To explore the relationships between water addition and total biomass, reproductive biomass, vegetative biomass, and R: T of each species, curve estimations were conducted, where linear, quadratic, power and exponential curves were tested. A better estimation has a smaller AIC (akaike information criterion) and a significant *P*-value (Cottingham et al., 2005). Log-transform was done when necessary. All statistics were done with SPSS 21.0.

## Results

**FIGURE 2:**
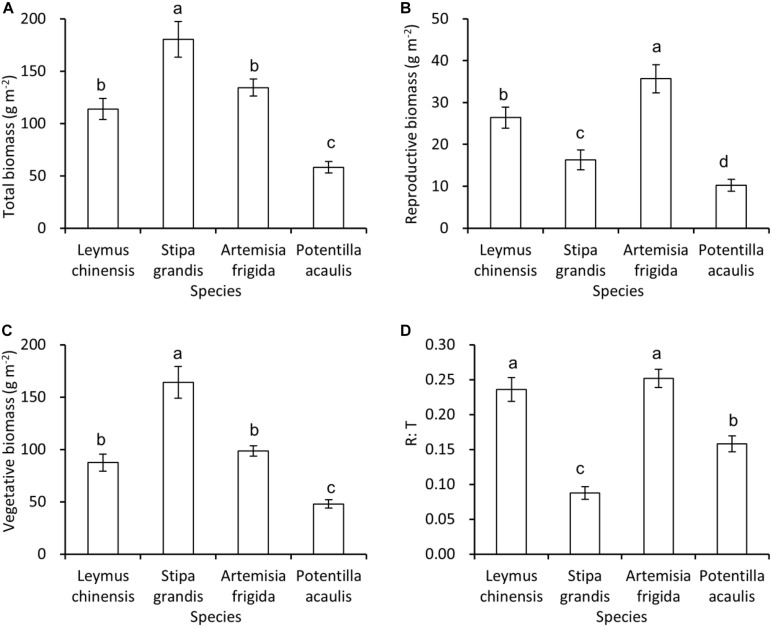
Mean ± SE of total biomass **(A)**, reproductive biomass **(B)**, vegetative biomass **(C)**, and reproductive: total biomass ratio (R: T) **(D)** as a function of species, i.e., *Leymus chinensis*, *Stipa grandis*, *Artemisia frigida*, and *Potentilla acaulis.* Significant (*P* < 0.05) differences between species have different letters (*post hoc* analyses with Bonferroni corrections).

Generally, *S. grandis* had the highest total biomass, and then followed by *L. chinensis* and *A. frigida*, and *P. acaulis* had the lowest total biomass (Figure 2A), and the same pattern was found in the vegetative biomass (Figure 2C). For the reproductive biomass, *A. frigida* was the highest, and then followed by *L. chinensis*, *S. grandis*, and *P. acaulis* (Figure 2B). Interestingly, *L. chinensis* and *A. frigida* had the highest R: T ratio, and then it was *P. acaulis*, while *S. grandis* had the lowest R: T ratio (Figure 2D).

**FIGURE 3:**
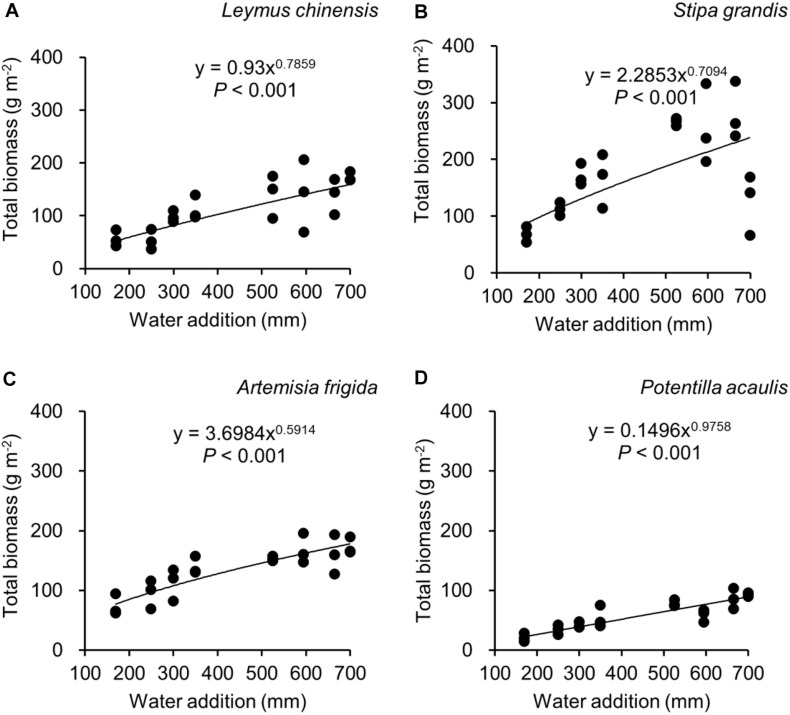
Regressions between water addition and total biomass, separately for *Leymus chinensis* **(A)**, *Stipa grandis* **(B)**, *Artemisia frigida* **(C)**, and *Potentilla acaulis* **(D)**.

**FIGURE 4:**
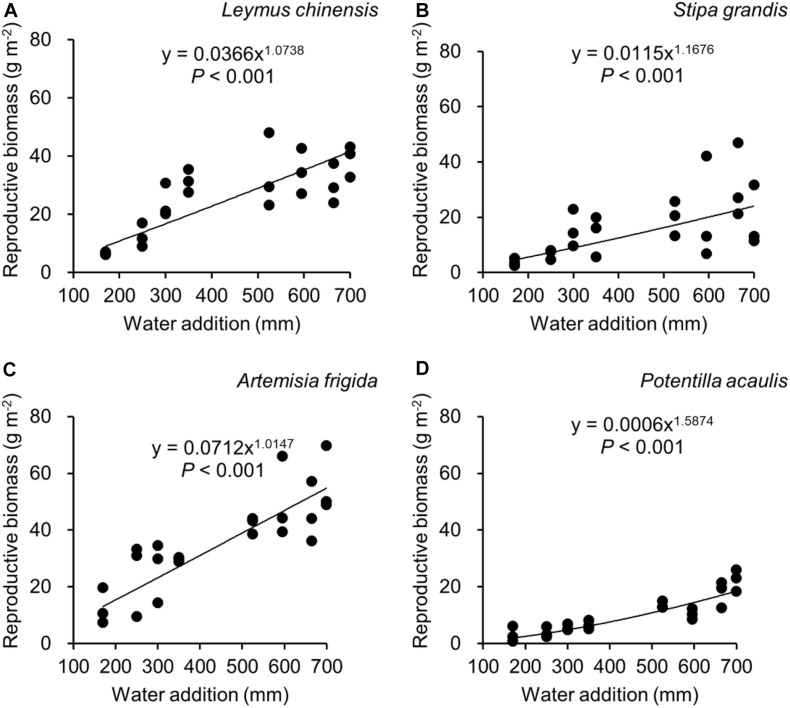
Regressions between water additions and reproductive biomass, separately for *Leymus chinensis* **(A)**, *Stipa grandis* **(B)**, *Artemisia frigida* **(C)**, and *Potentilla acaulis* **(D)**.

**FIGURE 5:**
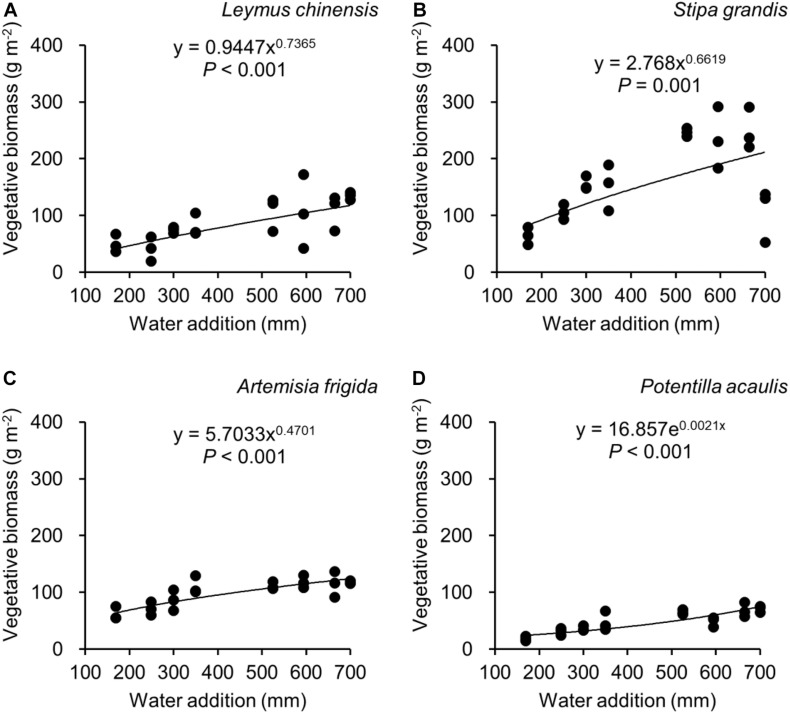
Regressions between water addition and vegetative biomass, separately for *Leymus chinensis* **(A)**, *Stipa grandis* **(B)**, *Artemisia frigida* **(C)**, and *Potentilla acaulis* **(D)**.

**FIGURE 6:**
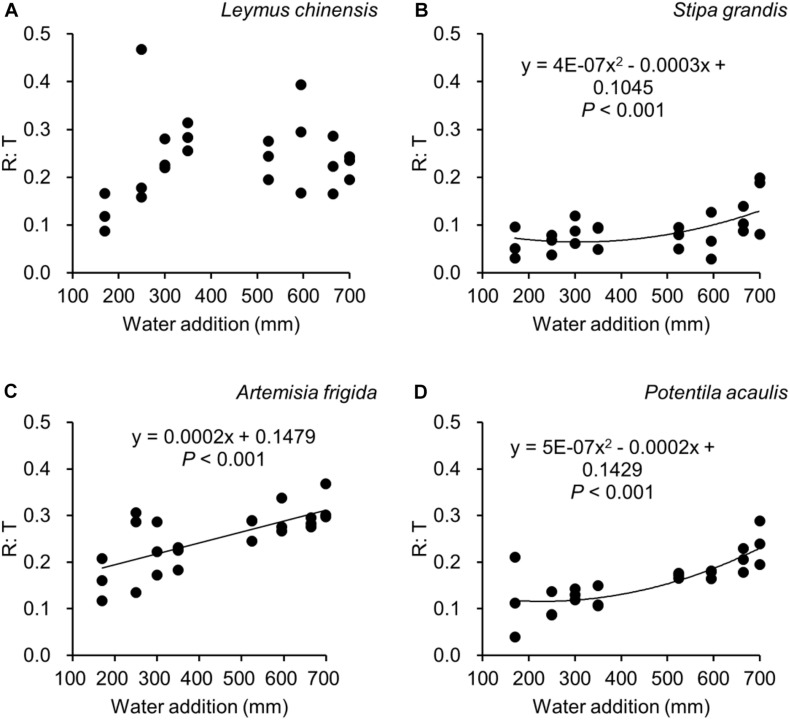
Regressions between water additions and R: T (i.e., the ratio of reproductive biomass and total biomass), separately for *Leymus chinensis* **(A)**, *Stipa grandis* **(B)**, *Artemisia frigida* **(C)**, and *Potentilla acaulis* **(D)**.

Water addition significantly affected the total biomass, reproductive biomass, vegetative biomass, and the ratio of reproductive biomass and total biomass (R: T), which were modified by species (Table 2 and Figures 3–6). In other words, interaction effects between species and water addition on vegetative biomass and total biomass were found. Thus, the relationships between water addition and these parameters were described separately for each species to better explore the different patterns.

**TABLE 2:** Effects of species, water addition and their interaction in two-way ANOVA on total biomass, reproductive biomass, vegetative biomass and ratio of reproductive biomass and total biomass (R: T), where significant differences are indicated in bold.

	Total biomass			Reproductive biomass		
		
	df	*F*	*P*	df	*F*	*P*
Species	3,64	73.2	**<0.001**	3,64	47.8	**<0.001**
Water addition	7,64	24.4	**<0.001**	7,64	18.2	**<0.001**
Species × Water addition	21,64	3.8	**<0.001**	21,64	1.5	0.122

	**Vegetative biomass**			**R: T**		
		
	**df**	***F***	***P***	**df**	***F***	***P***

Species	3,64	98.0	**<0.001**	3,64	45.2	**<0.001**
Water addition	7,64	22.0	**<0.001**	7,64	4.8	**<0.001**
Species × Water addition	21,64	5.0	**<0.001**	21,64	1.2	0.291

With increasing water addition all species showed a positive pattern in the relationships between water addition and total biomass (Table 3 and Figure 3), reproductive biomass (Table 4 and Figure 4) and vegetative biomass (Table 5 and Figure 5). Surprisingly, different patterns occurred in the relationship between water addition and R: T ratio for the four grasses species, where increasing water addition did not significantly impact the ratio of *L. chinensis* (Table 6 and Figure 6A), while it increased the ratio of *A. frigida* (Figure 6C), and it first decreased and then increased the ratio after reaching a peak in *S. grandis* and *P. acaulis* (Figures 6B,D), where the thresholds of water addition for *S. grandis* and *P. acaulis* were 375 and 200 mm, respectively. Note that a greater value of the R: T ratio indicates greater biomass investment in the reproductive organs.

**TABLE 3:** Results of the curve estimation of the relationship between water addition and total biomass, vegetative biomass, reproductive biomass and R: T ratio (i.e., the ratio of reproductive biomass and total biomass) of *Leymus chinensis* with linear, quadratic, power and exponential equations, where AIC, F, df, and *P*-value were showed, and significant differences are indicated in bold.

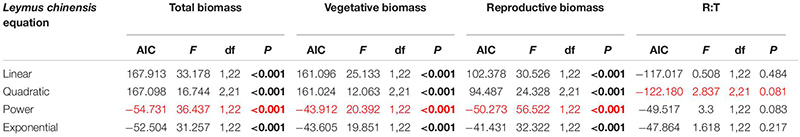

*A smaller AIC (akaike information criterion) with a significant P-value is a better estimation, which is marked in red.*

**TABLE 4:** Results of the curve estimation of the relationship between water addition and total biomass, vegetative biomass, reproductive biomass, and R: T ratio (i.e., the ratio of reproductive biomass and total biomass) of *Stipa grandis* with linear, quadratic, power and exponential equations, where AIC, F, df, and *P*-value were showed, and significant differences are indicated in bold.

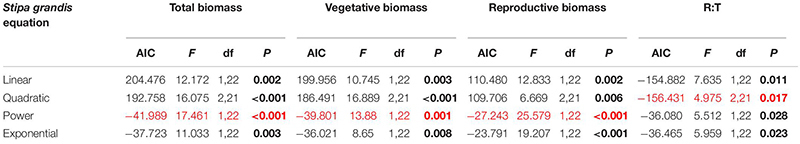

*A smaller AIC (akaike information criterion) with a significant P-value is a better estimation, which is marked in red.*

**TABLE 5:** Results of the curve estimation of the relationship between water addition and total biomass, vegetative biomass, reproductive biomass, and R: T ratio (i.e., the ratio of reproductive biomass and total biomass) of *Artemisia frigida* with linear, quadratic, power and exponential equations, where AIC, F, df, and *P*-value were showed, and significant differences are indicated in bold.

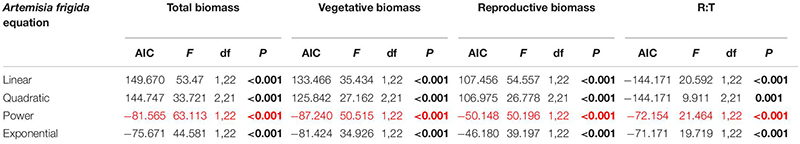

*A smaller AIC (akaike information criterion) with a significant P-value is a better estimation, which is marked in red.*

**TABLE 6:** Results of the curve estimation of the relationship between water addition and total biomass, vegetative biomass, reproductive biomass, and R: T ratio (i.e., the ratio of reproductive biomass and total biomass) of *Potentilla acaulis* with linear, quadratic, power and exponential equations, where AIC, F, df, and *P*-value were showed, and significant differences are indicated in bold.

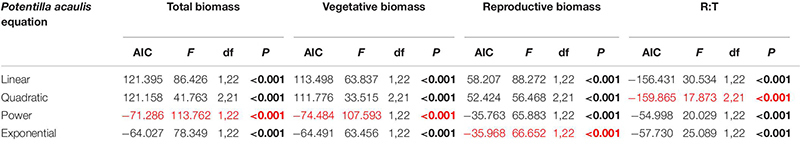

*A smaller AIC (akaike information criterion) with a significant P-value is a better estimation, which is marked in red.*

## Discussion

In this study, we found that plant species with rhizomes and stolons (i.e., *A. frigida*, *L. chinensis*, and *P. acaulis*) had a larger R: T ratio than that of species *S. grandis*. Moreover, water addition affected the reproductive allocation of plants, which was modified by species. Interestingly, increasing water availability did not always increase the reproductive allocation, suggesting different grasses species adopt different reproductive allocation strategies to adapt to the water additions.

Our first hypothesis, that species with a rhizomatous or stoloniferous growth form had low values of reproductive allocation, was not supported. We found that the reproductive value (i.e., R: T ratio in this case) of *S. grandis* was lower than that of species with rhizomes and stolons (i.e., *L. chinensis*, *A. frigida*, and *P. acaulis*), contrast with the finding of Wilson and Thompson (1989). Such differences may be derived from the different calculation of reproductive value, where it refers to the “weight of reproductive structures as a proportion of total aboveground biomass” in Wilson and Thompson (1989), while we adopted R: T ratio as the reproductive value, and reproductive allocation in our case refers to the biomass allocation between reproductive organs and vegetative organs during plant growth. However, similar trend with our original result was found when applying the method of Wilson and Thompson (1989) to calculate the reproductive value of the four grasses (Supplementary Figure S2 and Supplementary Table S1). Thus, such difference may be due to species and growing conditions (Harper and Ogden, 1970; Weiner, 2004). Further studies are needed to explore the underlying mechanisms of such patterns.

Our second hypothesis was that the reproductive allocation value of species with rhizomes and stolons (i.e., *L. chinensis, A. frigida*, and *P. acaulis*) may increase with increasing water availability, while a different pattern may occur in *Stipa grandis.* Our hypothesis was partly supported, where the reproductive allocation value of *A. frigida* indeed linearly increased with water addition (Figure 6C), and non-linear pattern between water addition and R: T ratio was indeed found in *Stipa grandis* (Figure 6B). However, the other two species with rhizomes and stolons (*L. chinensis* and *P. acaulis*) did not follow our expectation, where the reproductive allocation of *L. chinensis* was not affected by water additions, suggesting that the reproductive allocation of this species was not sensitive to the changes of water additions even though the total biomass and the reproductive biomass increased with increasing water availability (Figures 3A, 4A). This is also consistent with previous studies that the ratio of resources to reproductive organs such as flowering and fruiting is generally constant for a given species (Cartica and Quinn, 1982). Remarkably, we found U-shaped relationships between water addition and reproductive allocation in *P. acaulis*, which can be explained by the unimodal pattern between water amount and precipitation use efficiency in grasslands on the Tibetan Plateau (Zhou et al., 2020), where more vegetative biomass was found in grasslands with the intermediate level of water (Hui and Jackson, 2005; Fiala et al., 2009; Post and Knapp, 2019). These unexpected patterns merit further investigation.

Reproductive allocation of *A. frigida* increased with water additions (Figure 6C), indicating that *A. frigida* allocated more biomass to reproductive organs with increasing water availability. Such pattern mainly derives from the similar changes of total biomass and reproductive biomass, and both of them increased with increasing water addition (Figures 3C, 4C). Plant productivity was enhanced with increasing water availability, which is in line with the findings in previous studies (Li et al., 2003; Heisler-White et al., 2008; Fu et al., 2010). Note that more water addition could increase water losses via runoff, evaporation or deep soil water percolation (Knapp et al., 2008), and result in decreasing water resource utilization for plant productivity (Felton et al., 2019). High soil moisture can further prevent soil organic matter decomposition (Yang et al., 2002), as a result reducing production due to nutrients loss. Such a unimodal pattern was also found in the study of Zhang et al. (2007), where *Potentilla reptans var. sericophylla* had the largest reproductive allocation value at the moderate nutrient concentration.

Differences between our results and findings in previous studies could be found. For example, along a transect from east to west in China, Wang et al. (2001) found that reproductive biomass of *L. chinensis* was higher in the middle of this transect than that in the rest of this transect by conducting a field observation, where the water availability decreased along this transect. In other words, the biomass allocation to reproductive organs of *L. chinensis* firstly increased and then decreased with increasing water availability, which is not in line with our findings. Such different results in their study and our case can be explained as follows: (1) Soil conditions are different. The soil applied in our case is homogeneous collected from the nearby field and well mixed, while soils along the field transect in their case were heterogeneous. Studies have found that heterogeneous soils affect seed germination, plants productivity and species diversity, and their responses to climate change (Liu et al., 2017, 2017a,b, 2019; Liu and Hou, 2020). (2) Growing conditions are different. In our case, plants grew in a constant condition under a rainout shelter, while in their case plants grew in nature with climate variation and complicated interactions of different factors. Such differences can cause significant difference between different studies.

To further improve our understanding of the plant performances under climate change, two issues merit further research. One is to detect the effects of water availability on the trade-off between asexual and sexual reproduction. Studies have found that water availability affects the reproductive modes of plants, and in one case increasing water availability decreased the biomass allocation to sexual reproduction of *Hedysarum leave*, while reduced water availability increased the biomass allocation to asexual reproduction (Zhu et al., 2007). *Iris hexagona* allocated less biomass to sexual reproduction when plants growing in high-salt condition (Van Zandt et al., 2003). The other is to explore the responses of plants to the jointed effects of different environmental factors (e.g., temperature, rainfall) since several factors of climate change such as temperature and rainfall tend to co-vary (Luo et al., 2008; Sala et al., 2012; Komatsu et al., 2019), which can further improve our understanding of effects of climate change on grasslands.

## Conclusion

Water addition significantly affected the reproductive allocation of plants, which is modified by plant species. Increasing water addition linearly increased the biomass allocated to the reproductive organs of species with rhizomes and stolons such as *A. frigida*, while it non-linearly impacted the reproductive value of other species such as *Stipa grandis*, where increasing water availability firstly decreased and then increased biomass allocation to reproductive organs of this species when it reached to a peak. These results offer theoretical support for policy makers in grassland management in order to keep grassland healthy and sustainable.

## Data Availability Statement

The original contributions presented in the study are included in the article/Supplementary Material, further inquiries can be directed to the corresponding author.

## Author Contributions

ZL designed the research. ZL and YL drafted the manuscript and contributed to the interpretation of the results. Both authors contributed to the article and approved the submitted version.

## Conflict of Interest

The authors declare that the research was conducted in the absence of any commercial or financial relationships that could be construed as a potential conflict of interest. The reviewer GW declared a shared affiliation, though no other collaboration, with ZL to the handling editor.
