# Supplementary material for: Effects of Water Addition on Reproductive Allocation of Dominant Plant Species in Inner Mongolia Steppe
Source: Front Plant Sci. 2020 Nov 16;11:555743. doi: 10.3389/fpls.2020.555743 (PMC7701291; doi:10.3389/fpls.2020.555743)
Supplement: Supplementary file 1 [file Table_1.pdf]

Supplementary Figure S1 The mean annual precipitation in the study field from 1980 to 2000

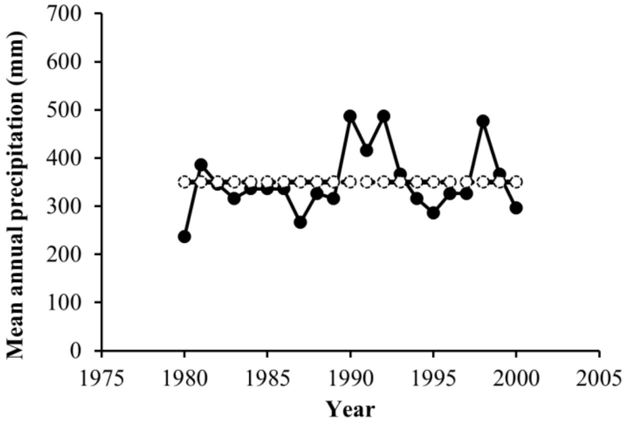

Supplementary Figure S2 Regressions between water additions and ratio of reproductive biomass to aboveground biomass (i.e. R: A), separately for *Leymus chinensis* (a), *Stipa grandis* (b), *Artemisia frigida* (c) and *Potentilla acaulis* (d)

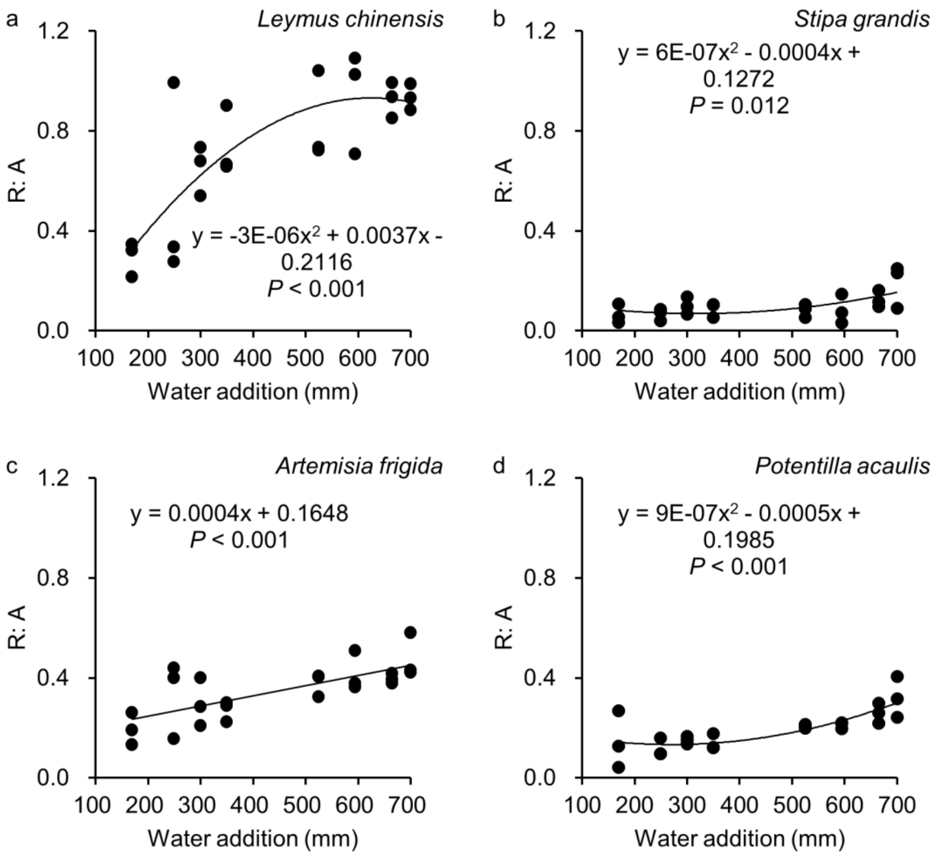

Supplementary Table 1 Results of the curve estimation of the relationship between water addition and ratio of reproductive biomass to aboveground biomass (i.e. R: A) of *Leymus chinensis*, *Stipa grandis*, *Artemisia frigida* and *Potentilla acaulis* with linear, quadratic, power and exponential equations, where AIC, F, df and *P* value were showed, and significant differences are indicated in bold. A smaller AIC (akaike information criterion) with a significant *P* value is a better estimation, which is marked in red

R: A	*Leymus chinensis*				*Stipa grandis*				*Artemisia frigida*				*Potentilla acaulis*			
Equation	AIC	F	df	*P*	AIC	F	df	*P*	AIC	F	df	*P*	AIC	F	df	*P*
Linear	-81.318	30.919	1,22	**<0.001**	-143.696	8.030	1,22	**0.010**	-119.289	21.906	1,22	**<0.001**	-138.246	28.294	1,22	**<0.001**
Quadratic	-85.891	20.058	2,21	**<0.001**	-146.172	5.516	2,21	**0.012**	-119.289	10.474	2,21	**0.001**	-142.772	18.478	2,21	**<0.001**
Power	-59.146	42.312	1,22	**<0.001**	-31.425	5.803	1,22	**0.025**	-62.145	23.738	1,22	**<0.001**	-46.695	18.866	1,22	**<0.001**
Exponential	-53.669	29.172	1,22	**<0.001**	-31.875	6.329	1,22	**0.020**	-60.963	21.563	1,22	**<0.001**	-49.987	24.886	1,22	**<0.001**
